# Synaptobrevin‐2 Containing Extracellular Vesicles Are Rapidly Incorporated Into Mammalian Neurons via a Dynamin‐Dependent Pathway

**DOI:** 10.1111/jnc.70551

**Published:** 2026-09-11

**Authors:** Victoria Hannett, A. Alejandro Vilcaes, Siewert Hugelier, Qing Tang, Melike Lakadamyali, Natali L. Chanaday

**Affiliations:** ^1^ Department of Physiology, Perelman School of Medicine University of Pennsylvania Philadelphia Pennsylvania USA; ^2^ Master of Biotechnology Program, School of Engineering and Applied Science University of Pennsylvania Philadelphia Pennsylvania USA; ^3^ Consejo Nacional de Investigaciones Científicas y Técnicas (CONICET) Universidad Nacional de Córdoba Córdoba Argentina; ^4^ Department of Biochemistry, Institute of Agriculture and Natural Resources, College of Arts and Sciences University of Nebraska‐Lincoln Lincoln Nebraska USA; ^5^ Penn Muscle Institute and Epigenetics Institute University of Pennsylvania Philadelphia Pennsylvania USA; ^6^ Department of Neuroscience, Perelman School of Medicine University of Pennsylvania Philadelphia Pennsylvania USA

**Keywords:** dynamin, endocytosis, extracellular vesicle, neuron, synaptic vesicle recycling, synaptobrevin‐2

## Abstract

Synaptobrevin‐2 (Syb2) is a SNARE protein essential for neurotransmitter release and communication in the nervous system. We previously showed that Syb2 is also exchanged among neurons via extracellular vesicles (EVs). Host neurons rapidly integrate exogenous Syb2 into their synaptic vesicle cycle to support neurotransmitter release. However, the endocytic mechanism by which neurons incorporate Syb2‐containing EVs is unknown. Here, we use a fusion of Syb2 with the pH‐sensitive GFP (Syb2‐pHluorin) to track the incorporation of Syb2‐containing EVs into rat hippocampal and cortical neurons and the trafficking of exogenous Syb2 to synaptic vesicles at synapses. We determined that Syb2‐containing EVs are endocytosed via a Dynamin‐dependent pathway, largely independently of macropinocytosis. The same endocytic route is used in the somatodendritic and axonal compartment and it occurs very rapidly, with the majority of Syb2‐pHluorin residing in internal acidic organelles at 30 min after EV addition, including synaptic vesicles at synapses. Leveraging this finding, we used EVs to sparsely deliver Syb2‐pHluorin to synapses and track the fusion and endocytosis of single synaptic vesicles. The results indicate that Syb2‐pHluorin‐positive synaptic vesicles are endocytosed with either ultrafast (< 1 s) or fast (~1–3 s) kinetics during synaptic transmission, suggesting limited diffusion and high fidelity in the fast retrieval of synaptic vesicle molecules immediately after fusion. Our findings expand our understanding of the mechanisms EVs use to enter neurons and open the door for future applications of EVs as vehicles to deliver fluorescent molecules in a neuron‐specific, targeted manner.

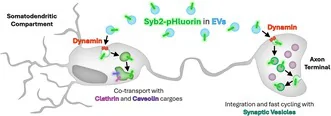

## Graphical abstract

In this article, Hannett and colleagues investigate extracellular vesicles (EVs) secreted by neurons, small membrane‐bound organelles that transmit information among cells. They found that EVs are mainly uptaken by target neurons via a mechanism dependent on the membrane scission protein Dynamin. This process occurs throughout the neuron, in the soma, dendrites, and axons. The authors also propose the use of EVs as a new delivery method to incorporate fluorescent protein into neurons, avoiding overexpression artifacts, and test an application to monitor the fusion‐endocytosis of synaptic vesicles.

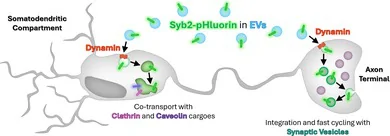

**Abbreviations**
Dex: Dextran‐Cy5.5
EVs: extracellular vesicles
LacCer: BODIPY‐lactosylceramide
MAP 2: microtubule‐associated protein 2
RRID: Research Resource Identifier (see scicrunch.org)
Syb2: Synaptobrevin‐2
Syn1: Synapsin‐1
Syp1: Synaptophysin‐1
Tf: Transferrin‐Alexa Fluor 64

## 1 Introduction

Neuronal communication spans a wide range of timescales and distances. Fast information processing in neuronal circuits is performed via electrical impulses and chemical transmission at synapses, while slower trophic and homeostatic signals are transferred via secretion of neuromodulator molecules and the latest added players, extracellular vesicles (Blanchette and Rodal 2020; Nieves Torres and Lee 2023; Sullivan et al. 2025). A common element regulating all these fast and slow communication modes is the vesicular protein Synaptobrevin‐2 (Syb2 or vesicle‐associated membrane protein 2—VAMP2). However, how Syb2 is transferred between the extracellular and intracellular compartments is unknown.

Syb2 was first discovered in synaptic vesicles from rat brain, where it is essential for the fusion of synaptic vesicles and subsequent release of neurotransmitters (Link et al. 1992). It was later discovered that Syb2 is a SNARE (soluble NSF attachment receptor) protein that, together with its target SNARE partners SNAP‐25 and Syntaxin‐1, catalyzes the fusion of vesicles with the target membrane (Hayashi et al. 1994; Pevsner et al. 1994). In this manner, Syb2 mediates the release of secretory vesicles and the surface delivery of plasma membrane proteins in multiple cell types, including neuronal, glial, endocrine, adipose and immune cells (Actis Dato et al. 2018; Bakr et al. 2021; Chang et al. 2015; Chen et al. 2023; Lam et al. 2022; Martin et al. 1998; Regazzi et al. 1995; Schoch et al. 2001; Uriarte et al. 2011). Syb2 has been proposed to be retrieved from the target membrane via multiple endocytic pathways, including Clathrin‐dependent and independent endocytosis (Cousin 2021; Gordon et al. 2011; Koo et al. 2015, 2011; Renard et al. 2015). Moreover, Syb2 couples exocytosis to endocytosis of synaptic vesicles during synaptic transmission, ensuring fast recycling of vesicles and maintaining the efficacy of neurotransmission (Chanaday and Kavalali 2021; Deák et al. 2004). Recently, Syb2 was found to be itself secreted to the extracellular space of neurons via extracellular vesicles (EVs), which could be a new non‐synaptic mechanism of neuron communication (Vilcaes et al. 2021).

Thus, Syb2 is involved in multiple forms of trafficking and secretion. However, the majority of previous research on Syb2 trafficking was performed by overexpressing fluorescently tagged versions of Syb2 in neurons, which causes abnormal subcellular distribution and recycling of Syb2, including higher plasma membrane levels and impaired endocytosis (Gordon et al. 2016; Pennuto et al. 2003). Here, we take advantage of our previous discovery showing Syb2 secretion via EVs and use these EVs to deliver tagged Syb2 to neurons. Using this novel approach, we investigate the endocytic mechanism of EVs carrying Syb2 into the neuron soma and axon and the transport of this exogenous Syb2 after incorporation. We also probe an application of this delivery approach to study the trafficking of Syb2, and hence of synaptic vesicles, at synapses in a more “native” environment. The advantages of this new delivery method are the absence of overexpression artifacts, low background fluorescence, and low toxicity—since transfection is not needed.

## 2 Methods

### 2.1 Dissociated Hippocampal and Cortical Cultures and Isolation of Extracellular Vesicles

Majority of the work was performed using postnatal Day 0–2 Sprague–Dawley rats (Charles River, SAS SD Strain Code 400) of both sexes. All hippocampi and cortices from the newborn pups in one litter were pulled together to generate one culture. For experiments using Dynamin triple knock‐out (Dnm TKO) neurons, we used a Dynamin3 knock‐out, Dynamin1/2 floxed mouse line previously generated by us (Dnm1^f/f^ Dnm2^f/f^ Dnm3 KO) (Afuwape et al. 2025). Postnatal Day 0 mice of both sexes were pulled together to generate one culture. Since this mouse line is a constitutive Dynamin3 KO, there is no wild‐type control in the same litter. For control experiments using SNAP25 KO neurons, embryonic Day 17–19 mice (SNAP25 KO) (Bronk et al. 2007) were used. In this case, each embryo was cultured individually and genotyped, to identify wild‐type, heterozygous and homozygous cultures, thus each independent culture had KO and control in the same setting. A total of 21 pregnant rats and 5 pregnant mice, and their respective litters, were used to generate the data presented here. All experiments were performed following protocols approved by the University of Pennsylvania Institutional Animal Care and Use Committee (Protocol #: 807365). Rodents were euthanized following the Humane Euthanasia of Laboratory Animals Standard Operating Procedures of the University of Pennsylvania. Specifically, dams were euthanized using a Crainey CO_2_ Chamber (secondary decapitation was performed to confirm death) and embryos and newborn pups were euthanized by decapitation.

Bilateral hippocampi and cortices were digested using 10 mg/mL trypsin and 0.5 mg/mL DNAse for 10 min at 37°C. Tissue was carefully dissociated using a P1000 pipette and cells were plated onto Matrigel (Corning, Cat. No. 354234) coated plastic bottom plates (for EVs isolation), glass coverslips (for electrophysiology), or 8‐well LabTek (for microscopy).

Neurons grown for microscopy experiments (EV recipient cells) were grown in basic growth medium consisting of MEM medium (no phenol red), 5 g/L D‐glucose, 0.2 g/L NaHCO3, 100 mg/L transferrin, 5% fetal bovine serum, 0.5 mM L‐glutamine, 2% B‐27 supplement, and 2–4 μM cytosine arabinoside. Cultures were kept in humidified incubators at 37°C and gassed with 95% air and 5% CO_2_.

Dissociated hippocampal neurons used for EV isolation were grown following our previously validated culture approach (Vilcaes et al. 2021). Briefly, neurons were maintained in Neurobasal (Gibco, Cat. No. 21103049) supplemented with 2% B‐27 (serum free, Gibco, Cat. No. 17504044), 1 mM sodium pyruvate, 2 mM glutamine, and 2 μM cytosine arabinoside.

For EVs isolation, neuron cultures at DIV 12–14 were used. 50% of culture media was replaced and 30% of fresh media was added every 24 h. After 60 h, culture supernatant was collected and cleared by serial centrifugations as previously described (Vilcaes et al. 2021). The resulting supernatant was used for EVs isolation by size exclusion chromatography using qEV1/35 mm columns from IZON (Cat. No. IC1‐35) and following the procedure suggested by the manufacturer and validated in our previous publication (Vilcaes et al. 2021). Nanoparticle Tracking Analysis (NTA) via ZetaView PMX220 Twin instrument (Particle Metrix) and negative stain electron microscopy were used to characterize isolated EVs (Figure S1).

**FIGURE 1:**
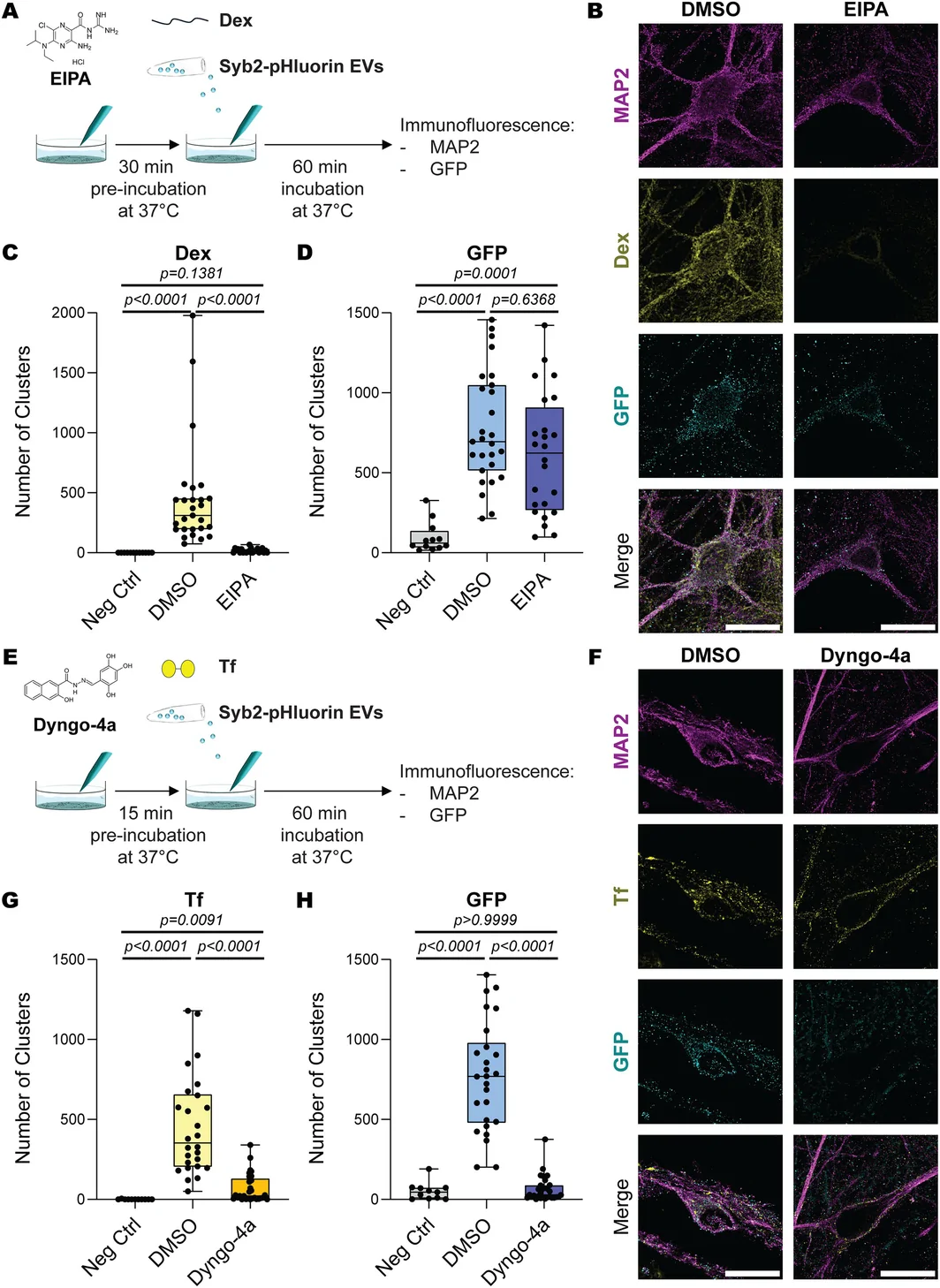
Syb2‐containing neuronal EV uptake in the somatodendritic compartment depends on Dynamin and occurs independently of macropinocytosis. (A) Experimental design. (B) Representative confocal images of neuronal cell bodies labeled with Dex (yellow), immunofluorescently labeled with anti‐MAP2 (magenta) and anti‐GFP (cyan). Z‐stacks of images are shown, presented as the maximum intensity projection of the volumetric data. White scale bars = 20 μm. (C, D) Number of Dex and GFP (EV‐delivered Syb2‐pHluorin) clusters in negative control, DMSO‐treated, and EIPA‐treated groups. (C) Kruskal–Wallis non‐parametric test, Kruskal–Wallis statistic = 50.83, *p* < 0.0001. (D) Kruskal*–*Wallis non‐parametric test, Kruskal–Wallis statistic = 27.15, *p* < 0.0001. (E) Experimental design. (F) Representative confocal images of neuronal cell bodies labeled with Tf (yellow), immunofluorescently labeled with anti‐MAP2 (magenta) and anti‐GFP (cyan). Z‐stacks of images are shown, presented as the maximum intensity projection of the volumetric data. White scale bars = 20 μm. (G, H) Number of Tf and GFP (EV‐delivered Syb2‐pHluorin) clusters in negative control, DMSO‐treated, and EIPA‐treated groups. (G) Kruskal*–*Wallis non‐parametric test, Kruskal–Wallis statistic = 49.51, *p* < 0.0001. (H) Kruskal*–*Wallis non‐parametric test, Kruskal–Wallis statistic = 29.31, *p* < 0.0001. For C, D and G, H: data are shown as box and whisker plots with each dot representing one individual neuron; results from Dunn's multiple comparisons test are displayed on the figure; data from 3 independent cultures.

**FIGURE 2:**
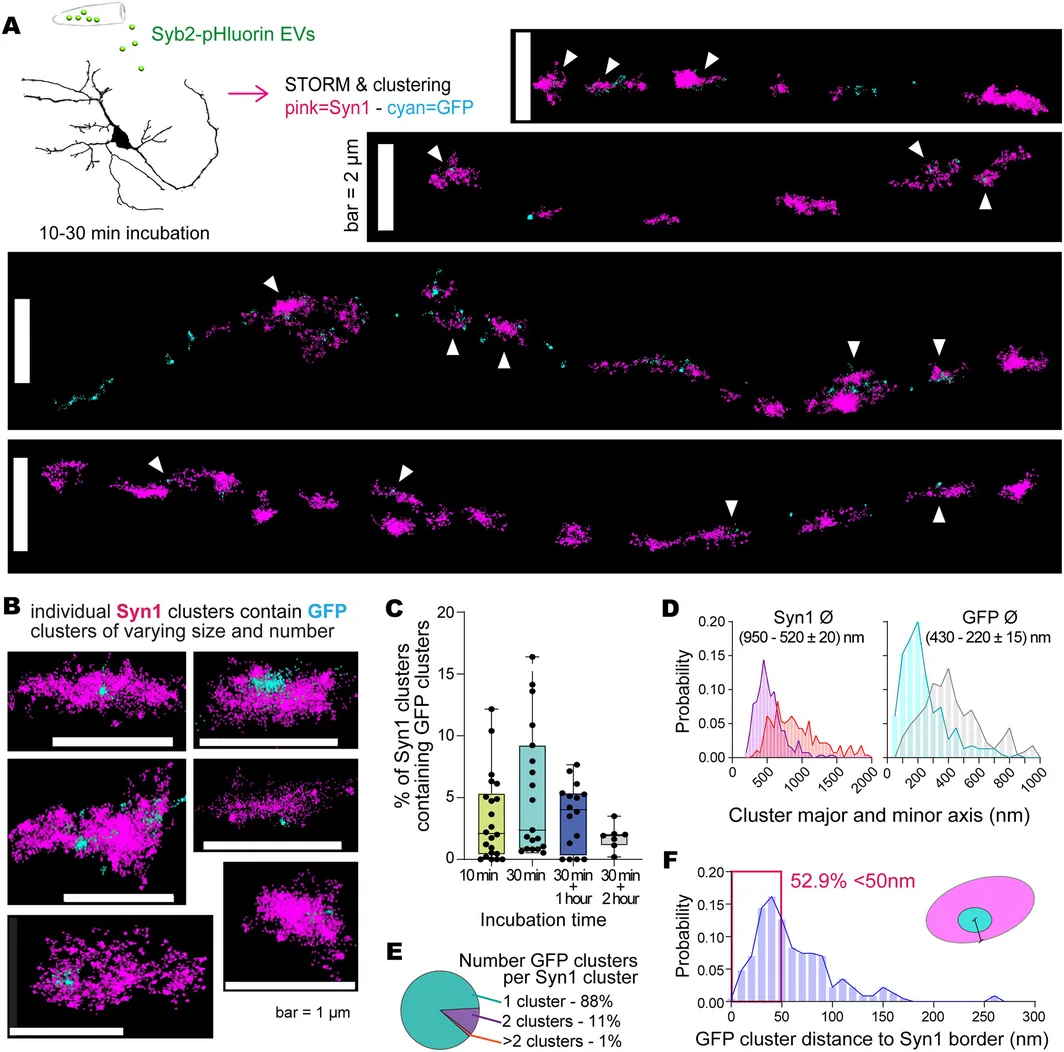
Syb2‐containing neuronal EVs are uptaken by axons. (A) Experimental design and representative STORM images showing Syn1 clusters in *en passant* axons (pink) and GFP clusters (corresponding to Syb2‐pHluorin, cyan). (B) Individual Syn1 presynaptic boutons containing GFP signal. (C) Percentage of Syn1 boutons containing GFP signal. Data is shown as box and whisker plots and each dot represents the average of all synapses from one image. (D) Diameters of Syn1 (left) and GFP (right) clusters. (E) Number of GFP clusters per Syn1 cluster. (F) Distance of GFP clusters to border of Syn1 cluster. Data from 3 independent cultures, 5–6 images were acquired for each.

**FIGURE 3:**
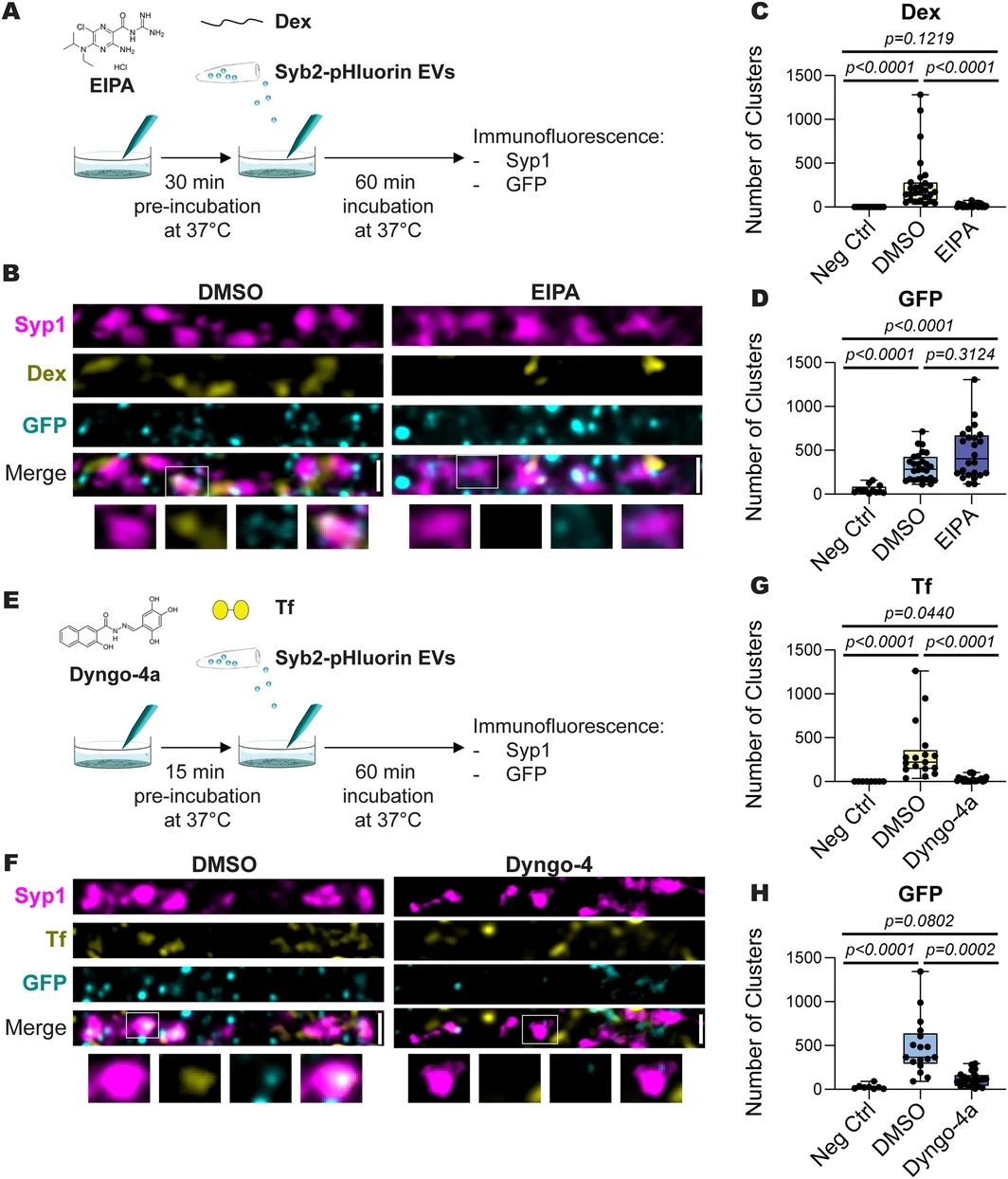
Syb2‐containing neuronal EV uptake in the axonal compartment is Dynamin‐dependent and occurs independently of macropinocytosis. (A) Experimental design. (B) Representative confocal images of synapses labeled with Dex (yellow), immunofluorescently labeled with anti‐Syp1 (magenta) and anti‐GFP (cyan). Z‐stacks of images are shown, presented as the maximum intensity projection of the volumetric data. White scale bars = 5 μm. (C, D) Number of Dex and GFP (EV‐delivered Syb2‐pHluorin) clusters in negative control, DMSO‐treated, and EIPA‐treated groups. (C) Kruskal‐Wallis non‐parametric test, Kruskal–Wallis statistic = 48.26, *p* < 0.0001. (D) Kruskal–Wallis non‐parametric test, Kruskal–Wallis statistic = 46.09, *p* < 0.0001. (E) Experimental design. (F) Representative confocal images of neuronal cell bodies labeled with Tf (yellow), immunofluorescently labeled with anti‐MAP2 (magenta) and anti‐GFP (cyan). Z‐stacks of images are shown, presented as the maximum intensity projection of the volumetric data. White scale bars = 5 μm. (G, H) Number of Tf and GFP (EV‐delivered Syb2‐pHluorin) clusters in negative control, DMSO‐treated, and EIPA‐treated groups. (G) Kruskal–Wallis non‐parametric test, Kruskal–Wallis statistic = 35.22, *p* < 0.0001. (D) Kruskal‐Wallis non‐parametric test, Kruskal–Wallis statistic = 30.51, *p* < 0.0001. For C, D and G, H: data are shown as box and whisker plots and each dot is the average of all presynaptic boutons quantified in one image; results from Dunn's multiple comparisons test are displayed on the figure; data from 3 independent cultures.

**FIGURE 4:**
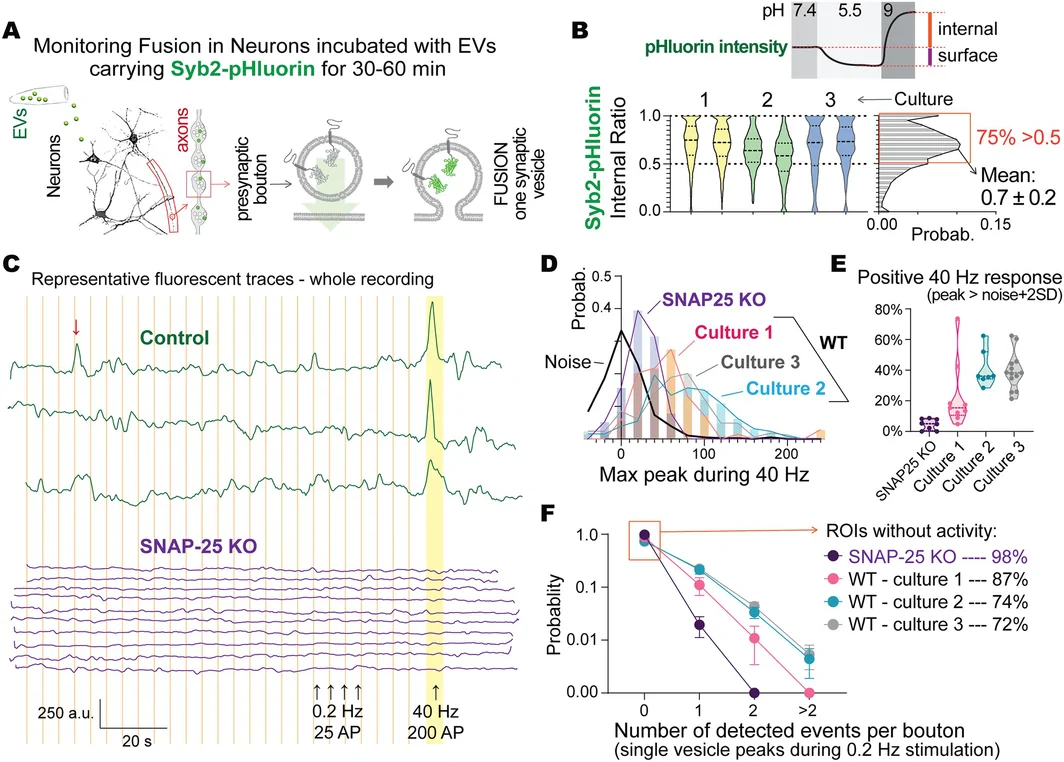
EVs sparsely deliver Syb2‐pHluorin to fusion‐competent presynaptic vesicles. (A) Experimental procedure. (B) Calculation of internal versus surface localization of Syb2‐pHluorin. (C) Fluorescence traces of control (WT) and SNAP‐25 KO synapses incubated with Syb2‐pHluorin EVs. (D) Histogram of maximal fluorescence amplitude after 40 Hz stimulation. Noise +2*SD was used as a threshold to define positive responses. (E) Percentage of synapses that responded to 40 Hz stimulation, based on the threshold set in C. (F) Probability of detection of single synaptic vesicle fusion events during low frequency 0.2 Hz stimulation. Each dot represents the average of all synapses from one imaging experiment, data from 3 independent cultures, 2–3 coverslips per culture, and one live imaging recording was performed per coverslip (same cultures were used for Figures 4 and 5). For B and E, violin plots are used to show the full distribution of all data, where the middle line shows the mean value and quartiles are represented by the top and bottom dotted lines. Since variability can be introduced by differences in neuron density among independent cultures and by efficacy of different EV batches, the data distribution of each independent experiment is shown separately to convey reproducibility of the biological effect.

**FIGURE 5:**
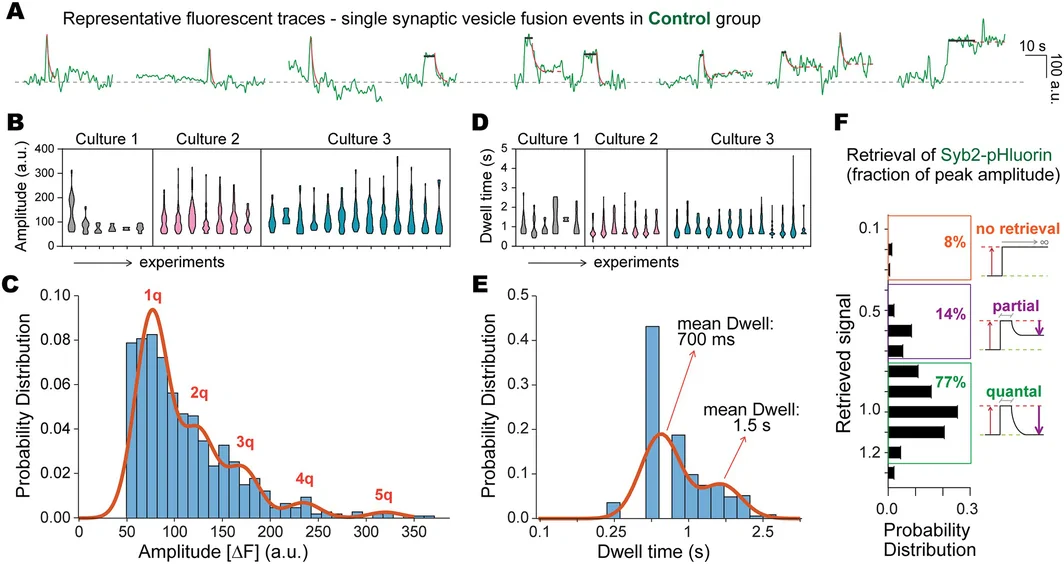
Newly fused Syb2 is rapidly and efficiently retrieved during synaptic activity. (A) Fluorescence traces of single vesicle fusion events. (B, C) Amplitudes and (D, E) Dwell times of single synaptic vesicle fusion events. Data from 3 independent cultures, 2 different batches of EV isolation were used in each culture. To fully convey reproducibility among independent experiments, the full distribution of values obtained for each coverslip and each culture is shown in B and D, and the compiled probability distribution of all experiments is plotted in C and E. For C and E, distributions were fitted with a Gaussian mixture model. (F) Retrieval of Syb2‐pHluorin, calculated as the ratio of total fluorescence decay after fusion over the event's amplitude.

For Figures 1, 2, 3, a concentration of 1 × 10^10^ particles/mL of EVs was used. For Figures 4 and 5, Figure S3 a concentration of 1 × 10^9^ particles/mL of EVs was used. These concentrations are in the range of normal concentration of EVs in the supernatant of dissociated neurons (Vilcaes et al. 2021).

### 2.2 Cloning and Lentiviral Infection

Lentivirus for Syb2‐pHluorin expression was produced in HEK293T cells (ATCC, Cat. No. CRL‐1573, RRID:CVCL_0045) by cotransfection of pFUGW‐Synaptobrevin2‐pHluorin vector (Ramirez et al. 2012) and 3 packaging plasmids (pCMV‐VSV‐G, pMDLg/pRRE, pRSV‐Rev—AddGene Cat. No. 8454, 12253 and 12251) (Dull et al. 1998; Stewart et al. 2003) using FuGENE 6 transfection reagent (Promega, Cat. No. E2692). Fresh, cleared supernatants containing lentiviruses were used for infection of days in vitro (DIV) 4 hippocampal neurons.

### 2.3 Incubation of Neurons With EVs, Endocytic Markers, and Endocytosis Inhibitors

For Airyscan experiments, neuron culture media for 14–22 DIV neurons was replaced with 100 μL of modified Tyrode's buffer (150 mM NaCl, 4 mM KCl, 10 mM glucose, 10 mM HEPES and 2 mM MgCl_2_, adjusted to pH 7.4 and 315‐320 mOsM) containing either 25 μM 5‐*N*‐ethyl‐*N*‐isopropyl‐amiloride (EIPA; MedChemExpress, Cat. No. HY‐101840) or 80 μM 3‐hydroxy‐2‐naphthalenecarboxylic acid (Dyngo‐4a; Abcam, Cat. No. ab120689) and pre‐incubated for 30 or 15 min at 37°C. For the positive controls (without endocytosis blocker), a similar volume of DMSO was added to the aforementioned mixture. Tyrode's buffer was replaced with 150 μL containing approximately 1.025 × 10^11^ particles/mL of EVs, 25 μM EIPA or 80 μM Dyngo‐4a, and 0.5 μg/mL 10 kDa Dextran‐Cy5.5 (Dex; MedChemExpress, Cat. No. HY‐D2460) or 16 μg/mL Transferrin‐Alexa647 (Tf; Invitrogen, Cat. No. T23366) and incubated for another 60 min at 37°C.

For Confocal experiments, neuron culture media for 14–22 DIV neurons was replaced with 120 μL of Neurobasal solution containing 7.5 × 10^8^ particles/mL of EVs, 16 μg/mL Tf, and 3.25 μg/mL BODIPY‐lactosylceramide (LacCer; Invitrogen, Cat. No. B34402) and incubated for 10, 30 or 60 min at 37°C in humidified incubators with 5% CO_2_.

### 2.4 Immunofluorescence

Neuron cultures were fixed with 1% PFA and 4% sucrose in DPBS and permeabilized using 0.01% digitonin in DPBS. After blocking with 3% BSA, primary antibodies against GFP (1:150) (Invitrogen, Cat. No. A11122, RRID:AB_221569), Syn1 (1:500) (Synaptic Systems, Cat. No. 106011, RRID:AB_10805139), Syp1 (1:500) (Synaptic Systems, Cat. No. 101006, RRID: AB_2622239), and/or MAP2 (1:1000) (Synaptic Systems, Cat. No. 188004, RRID:AB_2138181) were diluted in blocking buffer and incubated overnight at 4°C in a humid chamber. Secondary antibodies were incubated for 2 h at room temperature. For STORM, secondary antibodies were conjugated with the activator–reporter dye pairs (anti‐mouse conjugated to Cy3b and Alexa 405 at 1:100, anti‐rabbit conjugated to Alexa 647 and Alexa 405 at 1:100) by the laboratory of Dr. Melike Lakadamyali following previously reported procedures (Bates et al. 2007).

### 2.5 STORM Experiments

Three controls were performed in all independent experiments presented in Figure 2: A negative control consisting of dissociated neurons stained only with secondary antibodies (to control for secondary antibody specificity); a second negative control of cultures not treated with EVs and labeled with anti‐GFP antibody (to control for primary antibody specificity); and a positive control using neurons that overexpress Syb2‐pHluorin via lentiviral infection (necessary control to tune colocalization analysis).

STORM images were acquired with a STORM microscope from ONI (Nanoimager S) using a 100×, 1.4 NA oil‐immersion objective and a sCMOS Hamamatsu Orca Flash camera. To promote photoswitching of the fluorophores, a buffer containing 10 mM cysteamine MEA, 0.5 mg/mL glucose oxidase, 40 mg/mL catalase, and 10% glucose in PBS was used. The 640 and 561 lasers were used at 50% power, and the 405 laser was used at 5%–8% or 15% power, respectively. 30000 images were acquired with 10 ms exposure time for each channel (647 channel was always acquired first). STORM image localizations were obtained using Nanoimager software (ONI) and analyzed using custom‐written MATLAB codes. In brief, clustering of the localizations was performed using Voronoi tessellation, a widely used method that evaluates the local density of individual localizations or fluorophores and allows a precise and unbiased determination of object shape and density, and that does not require user input of predetermined parameters, for example, radius. The generated Voronoi cells or clusters represent one reconstructed biological object or structure. Voronoi segmentation was performed using the same threshold for all experiments (threshold was set using the probability density function of negative controls), and clusters with < 5 localizations were filtered out to eliminate noise and false positive data (so there were no clusters detected in the negative controls). The Syn1 clusters were used as ‘reference’ data for the colocalization analysis, and the GFP clusters as the ‘colocalization’ data (see https://github.com/melikelakadamyali/StormAnalysisSoftware). The code transforms the reference cluster into high definition ‘alphaShape’ objects and then considers a GFP cluster to be colocalized when there is at least 40% overlap between the GFP localizations and the reference cluster alphaShape object. Using these parameters, there was more than 85% overlap and colocalization between Syn1 clusters and GFP clusters in the positive control. For the GFP clusters that are colocalized, the distance metric reported in Figure 2F was calculated by determining the distance between the center of the colocalization cluster and the closest border point of the reference cluster.

Fiducial markers (1:10000 dilution) were added to each imaged sample and used to correct for drifting. Alignment of the two (Syn1 and GFP) channels was checked before performing colocalization analysis.

### 2.6 Airyscan and Confocal Microscopy

Super‐resolution imaging was performed using a ZEISS LSM 980 confocal microscope equipped with an Airyscan 2 detector (Zeiss) using a 63× (NA1.4) objective and a pixel size of 0.035 × 0.035 × 0.13 μm (x, y, z). Around 3–8 Z‐stacks were acquired in different regions of each coverslip/sample. To achieve increased spatial resolution, the acquired raw multi‐view data were processed using Airyscan Joint Deconvolution in the ZEN Blue software. The algorithm utilizes a spatially variant theoretical Point Spread Function and an iterative multi‐view Richardson‐Lucy deconvolution method to computationally resolve and constructively reassign the 32 individual detector element views (Prazeres da Costa et al. 2021). Deconvolution was run with 5 iterations at standard resolution, resulting in an effective resolution of 90 nm. Images were obtained and analyzed in a blind fashion: V.H. performed the experiments and the immunofluorescence staining, and V.H. collected images at the Airyscan confocal microscope being blind to treatment group.

To detect fluorescent objects in Airyscan images, segmentation was performed using Trainable Weka Segmentation (TWS) (Arganda‐Carreras et al. 2017). In this study, 3 classifiers were trained to identify Dex, Tf, and GFP. Classifiers were each trained on 3 images. Within TWS's settings, the training features Mean and Variance with a minimum sigma of 1.0 and a maximum sigma of 8.0 were selected. The FastRandomForest option was chosen as classifier. V.H. analyzed the images in an unbiased manner using macros and standardized pipelines in Fiji (Schindelin et al. 2012).

Confocal images were acquired using a Zeiss LSM 980 Confocal Microscope (Carl Zeiss) with a 63X (NA1.4) objective and a pixel size of 0.044 × 0.044 × 0.21 μm (x, y, z). Around 6–8 Z‐stacks (step size of 1/2 of the z resolution) were acquired in different regions of each coverslip/sample. Regions of interest (ROIs) were drawn around individual neurons (soma and dendrites) in each image using the MAP2 channel. Then, the Fiji/ImageJ plugin Distance Analysis (DiAna) (Gilles et al. 2017) was used to determine nearest‐neighbor distances between GFP (EV), LacCer, and Tf signals. In brief, DiAna Labelisation was used to identify individual fluorescent objects (clusters). Thresholds for DiAna Labelisation were determined using a negative control (neurons incubated with antibodies but without markers or EVs). Nearest‐neighbor distances were for all pairwise combinations of the analyzed markers using DiAna Distance Analysis. The data was then filtered to determine the number of positive objects that were within 250 nm distance compared to the total number of positive objects; this distance was defined as colocalization since it is the limit of resolution of our images. Images were obtained and analyzed in a blind fashion: V.H. performed the experiments and the immunofluorescence staining, A.A.V. collected images at the confocal being blind to treatment group, then V.H. analyzed the images in an unbiased manner using macros and standardized pipelines in Fiji (Schindelin et al. 2012).

### 2.7 Live Fluorescence Imaging

14–22 DIV cultured hippocampal neurons were imaged in modified Tyrode's buffer containing 150 mM NaCl, 4 mM KCl, 10 mM glucose, 10 mM HEPES and 2 mM MgCl2, adjusted to pH 7.4 and 315–320 mOsM. To avoid recurrent network activity, the following agonists against ionotropic glutamate receptors were added: 6‐cyano‐7‐nitroquinoxaline‐2,3‐dione (CNQX, 10 μM; Sigma‐Aldrich, Cat. No. C239) and aminophosphonopentanoic acid (AP‐5, 50 μM; Sigma‐Aldrich, Cat. No. A8054). Fluorescence was recorded using a Nikon Eclipse TE2000‐U microscope (Nikon) and an Andor iXon+ back‐illuminated EMCCD camera (Model no. DU‐897E‐CSO‐#BV). For illumination, we used a Lambda‐DG4 illumination system (Sutter Instruments) with a FITC emission filter. Images were acquired at 5 Hz. Circular regions of interest (ROI) of 2 μm diameter were drawn around local fluorescence maxima (putative presynaptic boutons) and measured using Fiji (Schindelin et al. 2012). Fluorescence peaks synchronous respect to the stimulation were detected and analyzed using MATLAB (Chanaday and Kavalali 2018, 2021).

### 2.8 Whole‐Cell Voltage‐Clamp Electrophysiology

Only pyramidal neurons were patched for whole cell recordings. Voltage was clamped at −70 mV using MultiClamp 700B and pCLAMP 11 software (Molecular Devices), filtering at 2 kHz and sampling at 10 kHz. Pipette resistance was ~2–5 MΩ and access resistance 5–20 MΩ for all experiments. The internal pipette solution contained 115 mM CsMeSO_3_, 10 mM CsCl, 5 mM NaCl, 10 mM HEPES, 0.6 mM EGTA, 20 mM tetraethylammonium chloride, 4 mM Mg‐ATP, 0.3 mM Na_2_GTP and 10 mM QX‐314 (lidocaine N‐ethyl bromide). The final solution was adjusted to pH 7.3 and 305–310 mOsM. The extracellular solution was a modified Tyrode's solution containing 150 mM NaCl, 4 mM KCl, 10 mM glucose, 10 mM HEPES and 2 mM MgCl_2_, adjusted to pH 7.4 and 315–320 mOsM. To isolate spontaneous inhibitory postsynaptic currents, antagonists of ionotropic glutamate receptors were added: 10 μM 6‐cyano‐7‐nitroquinoxaline‐2,3‐dione (CNQX) and 50 μM aminophosphonopentanoic acid (AP‐5); along with 1 μM tetrodotoxin (TTX) to block action potential firing. All recordings were performed at room temperature and analysis was performed using Clampfit (pClamp 11).

### 2.9 Statistical Analysis

The approach we used for this research is hypothesis‐free; we did not have prior knowledge of the type of result or values we were going to obtain or of the magnitude of the effect of extracellular vesicles. For that reason, prior statistical power analysis and sample‐size estimation was not possible. Nevertheless, the number of experiments and samples needed can be inferred from previous, similar experiments performed by our lab (Chanaday and Kavalali 2018, 2021; Vilcaes et al. 2021). Assuming the reproducibility of experimental conditions and settings, we estimated that a minimum of 2 independent cultures with 2 coverslips per experimental group was enough for significance testing and finding the presence or absence of differences or tendencies among groups. Based on the variances and tendencies observed, in some cases the addition of extra experiments was decided.

For all figures, the full distribution of all experimental data is shown as box and whisker plots where the whiskers show the full distribution of the data, box height represents quartiles and the middle line represents the median value. Statistical analysis was performed using GraphPad Prism software (version 8.0.2). Normality was tested first and, based on the result, parametric or non‐parametric statistics were applied. All data points were included in the analysis (no test for outliers was conducted). For Figures 1 and 3, data were analyzed using Kruskal‐Wallis non‐parametric tests with Dunn's multiple comparisons tests; statistical details are provided in the figures and their legends. For Figure 5, histograms were fitted in MATLAB using a Gaussian mixture model with one to twenty components, and the model with the smallest Bayes information criterion (BIC) value was automatically selected as the best fit (Chanaday and Kavalali 2021). All data results from 2 to 3 independent cultures. Efforts were made to minimize the number of animals used for the experiments.

## 3 Results

### 3.1 Uptake of Syb2‐Containing EVs Into the Somatodendritic Compartment of Neurons Is Dynamin‐Dependent and Occurs Largely Segregated From Macropinocytosis

We previously showed that dissociated hippocampal neurons secrete Syb2 via EVs through a mechanism that depends on the tetraspanin CD81 (Vilcaes et al. 2021). Using EVs isolated from neurons expressing Syb2 tagged with pH‐sensitive GFP—pHluorin—we found that neuronal EVs containing Syb2‐pHluorin are preferentially internalized into neurons (Vilcaes et al. 2021), although the uptake mechanism is unknown. The preferred uptake pathways for EVs in multiple cell types seem to be endocytosis (Mulcahy et al. 2014). Endocytic pathways can be globally classified depending on their reliance on the membrane scission molecule Dynamin (Gundu et al. 2022; Rennick et al. 2021). Various endocytic pathways have been implicated in EV endocytosis in other cell types, including Dynamin‐dependent pathways—Clathrin‐mediated endocytosis and Caveolin‐dependent endocytosis—and Dynamin‐independent pathways—macropinocytosis and phagocytosis—with many studies suggesting coexistence of multiple pathways (Mulcahy et al. 2014). For neurons in particular, a previous report suggested that EVs' incorporation into cortical neurons occurs via Clathrin‐ and Dynamin‐dependent endocytosis (Solana‐Balaguer et al. 2023). Therefore, to determine the internalization route of Syb2‐pHluorin‐containing EVs into the somatodendritic compartment of dissociated neurons, we used the specific inhibitors EIPA and Dyngo‐4a, which block macropinocytosis and Dynamin‐dependent endocytosis, respectively.

Syb2‐containing EVs were isolated from the media of astrocyte‐free primary neuron cultures using size exclusion chromatography (qEV Exosome Isolation from IZON) and characterized using nanoparticle tracking analysis (NTA) and transmission electron microscopy (Figure S1A,B) as previously described (Vilcaes et al. 2021). Functionality of the EVs was further corroborated by an increase in the frequency of spontaneous inhibitory neurotransmission (Figure S1C,D) as previously reported (Vilcaes et al. 2021). After pre‐treating 14–22 DIV neurons with 25 μM EIPA or 80 μM Dyngo‐4a, we treated with Dextran‐Cy5.5 (Dex)—a macropinocytosis marker—or Transferrin‐Alexa647 (Tf)—a classical marker for Clathrin‐mediated endocytosis—and incubated for another 60 min at 37°C. We then immunolabeled the neurons with GFP—to detect Syb2‐pHluorin molecules delivered via EVs—and microtubule‐associated protein 2 (MAP2)–to visualize the somatodendritic compartment, and performed Airyscan microscopy (Figure 1A,D). Combined (joint) deconvolution was performed on the images to increase the image resolution (Prazeres da Costa et al. 2021), and individual cell bodies (or soma) and dendrites were selected for analysis. Fluorescent puncta or clusters in deconvolved images were segmented and classified using the Fiji plugin Trainable Weka Segmentation 3D (Arganda‐Carreras et al. 2017). While pretreatment with EIPA significantly reduced the endocytosis of Dex into the somatodendritic compartment of neurons, it did not significantly affect the internalization of EV‐delivered Syb2‐pHluorin (detected with anti‐GFP antibody; Figure 1B–D). Conversely, pretreatment with Dyngo‐4a abolished the endocytosis of EV‐delivered Syb2‐pHluorin, as well as Tf (Figure 1F–H). This suggests that Syb2‐containing EV uptake is dependent on Dynamin.

To further elucidate the trafficking dynamics of Syb2‐containing EVs after Dynamin‐dependent internalization, we quantified the colocalization of GFP with Tf and BODIPY‐lactosylceramide (LacCer), markers of Cathrin‐mediated and Caveolin‐dependent endocytosis, respectively. We incubated 14–22 DIV neurons with Syb2‐pHluorin EVs, Tf, and LacCer for 10, 30, or 60 min at 37°C, then performed confocal microscopy (Figure S2A,B). Images were segmented and analyzed using the Fiji plugin DiAna (Gilles et al. 2017) to determine the nearest‐neighbor distance between the different endocytic intermediaries. Objects closer than 250 nm were defined as colocalizing. We observed endocytosis and partial colocalization of Tf and GFP at 10 min post‐addition (Figure S2C), but negligible intracellular content of LacCer, suggesting that Caveolin‐mediated uptake may be slower in neurons than Clathrin‐mediated. Proximity among all three markers increased as a function of time (Figure S2C), indicating that all Dynamin‐dependent cargoes converge to the same late endosomes and/or trafficking intermediaries for posterior sorting. Thus, this measurement points to Syb2‐containing EVs likely being transported via classical endosomal pathways after endocytosis, coinciding with other Dynamin‐dependent cargoes.

### 3.2 Syb2‐Containing EVs Are Directly Internalized Into Axons via a Dynamin‐Dependent Pathway

We previously reported that Syb2‐containing EVs increase spontaneous inhibitory neurotransmission in the target neuron as soon as 20–30 min after addition (Vilcaes et al. 2021). The data presented here also suggest that, after internalization into the neuron soma, Syb2 from the EVs is trafficked through an endosomal pathway that converges with Clathrin and Caveolin cargoes 30–60 min after internalization. That timescale of intracellular transport is incompatible with the fast functional effects in synaptic transmission we previously uncovered, suggesting that Syb2‐containing EVs may be directly endocytosed at the synapse. Due to the narrow caliber of axons (< 1 μm wide), we turned to super‐resolution microscopy to determine the localization of EVs‐delivered Syb2‐pHluorin along axons of dissociated hippocampal neurons. 14–22 DIV dissociated neurons were incubated with EVs for 30 min at 37°C, then fixed, immunostained, and imaged via Stochastic Optical Reconstruction Microscopy (STORM; Figure 2). Exogenous Syb2‐pHluorin, incorporated into neurons from the EVs, was identified with anti‐GFP antibody. Image segmentation was performed using a previously validated Voronoi tessellation method (Gyparaki et al. 2021) (clustering and colocalization parameters were determined using negative and positive controls, see Methods for details). In EV‐treated neurons, small GFP‐positive clusters were observed in structures resembling *en passant* axons (Figure 2A), usually colocalizing with the presynaptic marker Synapsin‐1 (Syn1; Figure 2A,B). Only a small fraction of presynaptic terminals contained GFP (< 10%–15%; Figure 2C), agreeing with our previous measurements using confocal microscopy (Vilcaes et al. 2021). To avoid detection of non‐specific signal, clusters containing less than 5 localizations were filtered out of the resulting STORM images (this limit was determined using negative controls). Therefore, our method is less sensitive to detect single GFP molecules compared to GFP clusters, so it is likely that we are underestimating the real number of presynaptic terminals containing exogenously added Syb2‐pHluorin. The number of Syn1‐positive presynaptic boutons containing GFP clusters had already reached a peak at 10–30 min after EVs addition and did not further increase over time (Figure 2C), suggesting that Syb2‐containing EVs are incorporated directly into the synapse and that there is little to no contribution of extra Syb2‐pHluorin being transported from the soma in the timescale of 10 min–2 h.

Exogenous Syb2, added via EVs, augments inhibitory neurotransmission by increasing the propensity of synaptic vesicles to fuse (Vilcaes et al. 2021). So next we asked whether the presynaptic clusters of GFP represented endosomes or synaptic vesicles. To answer this question, we evaluated the number, size, and spatial distribution of GFP clusters. Average minor and major axes (diameter) of Syn1 clusters were 0.5 and 1 μm, respectively (Figure 2D), supporting that segmented clusters correspond to *bona fide* presynaptic boutons. Syb2‐pHluorin (GFP) clusters were smaller, with mean minor and major axes of 0.2 and 0.4 μm (Figure 2D). Due to the small size and high density of synaptic vesicles, combined with the artifacts in determining size when using antibodies for detection, we cannot differentiate if larger Syb2‐pHluorin (GFP) clusters represent groups of synaptic vesicles or small recycling endosomes, similar to previously described biogenic precursors (Régnier‐Vigouroux et al. 1991; Watson et al. 2023). However, 6% of Syb2‐pHluorin clusters had both diameters smaller than 100 nm, suggesting the detection of putative individual synaptic vesicles. Most (88%) of the Syn1 presynaptic boutons only contained one GFP cluster (Figure 2E), which was found in close proximity to the bouton surface (defined as the distance to the border of the Syn1 cluster; 52.9% at < 50 nm and ~45% at < 150 nm; Figure 2F). This distribution resembles the distribution of synaptic vesicles, where the highest density was found closer to the active zone membrane (Schikorski 2014). In summary, using STORM, we uncovered that Syb2‐pHluorin incorporated from exogenously added EVs is localized to small vesicles in close proximity to the presynaptic plasma membrane, possibly ready to fuse in response to neuron activity.

Given that neurons are highly polarized into two distinct structural and functional domains—the somatodendritic and axonal compartments (Lasiecka and Winckler 2011), we next investigated whether Syb2‐containing EVs use the same or separate endocytic mechanisms in presynaptic axonal boutons as in the somatodendritic compartment. We repeated treatment with inhibitors of macropinocytosis and Dynamin‐dependent endocytosis, this time labeling presynaptic boutons with Synaptophysin‐1 (Syp1). We pre‐treated 14–22 DIV neurons with 25 μM EIPA or 80 μM Dyngo‐4a, added EVs and Dex or Tf for another 60 min at 37°C, and performed Airyscan microscopy (Figure 3A,E). Images were acquired, deconvolved, and segmented using the same algorithms described above (data in Figure 1). Similar to our findings in the somatodendritic compartment, pretreatment with EIPA significantly reduced the endocytosis of Dex but not of EV‐delivered Syb2‐pHluorin (Figure 3B–D), while pretreatment with Dyngo‐4a abolished the endocytosis of both Tf and EV‐delivered Syb2‐pHluorin (Figure 3F–H). These results indicate that Syb2‐containing EVs are endocytosed into both the somatodendritic and axonal compartments via a Dynamin‐dependent pathway.

We previously showed that EV‐delivered Syb2 is rapidly incorporated into functional synaptic vesicles, since it increases the spontaneous fusion of vesicles at inhibitory synapses, it can partially rescue neurotransmission in Syb2 knock‐out neurons and it depends on Syb2's partner SNARE, SNAP25 (Vilcaes et al. 2021). To test whether this process is dependent on Dynamin, we performed whole‐cell voltage‐clamp electrophysiology on 20–25 DIV hippocampal neurons lacking all three Dynamin isoforms (Afuwape et al. 2025) (Figure S3). We observed no statistically significant changes in either the frequency or the amplitude of miniature inhibitory post‐synaptic currents (mIPSC) in response to the addition of Syb2‐containing EVs in Dynamin triple knock‐out neurons (Figure S3B,C), suggesting that Dynamin is necessary for the previously observed EV‐mediated increase in spontaneous inhibitory neurotransmission (also compare with Figure S1C,D). Collectively, the experiments presented so far support a mechanism where EV‐delivered Syb2 is directly endocytosed into axons via a Dynamin‐driven pathway and posteriorly integrated into the synaptic vesicle cycle.

### 3.3 EVs as a New Method to Deliver Syb2‐pHluorin to Presynaptic Terminals

Our confocal and STORM experiments suggested that most of EV‐delivered Syb2‐pHluorin molecules resided in internal organelles, so we next corroborated this observation by measuring the ratio of pHluorin within acidic organelles compared to the neuronal plasma membrane. Surface and total fluorophore levels were measured by quenching with acidic buffer of pH = 5.5 or perfusing 50 mM NH_4_Cl pH = 9 solution, respectively (Figure 4A,B; also see (Sankaranarayanan et al. 2000)). The ratio of internal Syb2‐pHluorin was then estimated by subtracting surface signal from total fluorescence intensity (Figure 4B). The majority (> 75%) of Syb2‐pHluorin incorporated into neurons from EVs resided internally, possibly in trafficking organelles (Figure 4A,B). Taken together with the STORM measurements, these results indicate that presynaptic boutons only received few copy number of Syb2‐pHluorin molecules that were mostly located in one internal trafficking organelle close to the presynaptic plasma membrane. To test whether these organelles are fusion competent, we stimulated 14–22 DIV neurons at low and high frequency to monitor activity‐dependent exo‐endocytosis of EV‐delivered Syb2‐pHluorin (Figure 4A,C; also see (Chanaday and Kavalali 2018, 2021)). As a negative control and threshold for detection of fusion events, we used 14–22 DIV dissociated neurons from SNAP‐25 knock‐out (KO) mice, which have almost complete loss of neurotransmission (Figure 4C). As expected, less than 10% of SNAP‐25 KO presynaptic boutons responded to high frequency stimulation (train of 200 action potentials—APs—at 40 Hz), while 20%–40% of wild‐type (WT) synapses showed a peak in fluorescence corresponding to fusion and endocytosis of Syb2‐pHluorin (Figure 4D,E). Due to the low number of synapses and synaptic vesicles receiving exogenous Syb2‐pHluorin from EVs, probability of detecting fusion in response to single APs was very low (Figure 4F) but detectably larger in WT cultures compared to SNAP‐25 KO (Figure 4F). In some synapses, 2 or 3 individual fusion events were detected during the recording (Figure 4F), corroborating our STORM measurements that indicated larger or multiple clusters, that is, groups of more than one synaptic vesicle positive for Syb2‐pHluorin, in some presynaptic boutons. Thus, EVs sparsely deliver Syb2 directly to presynaptic boutons, leading to rapid (< 30 min) integration of exogenous Syb2 into a few fusion‐competent synaptic vesicles.

After catalyzing the release of secretory vesicles, Syb2 must be retrieved from the plasma membrane in order to facilitate successive rounds of exocytosis. Inefficient Syb2 retrieval thus leads to depression of neurotransmitter release and synaptic transmission during sustained neuron activity (Rajappa et al. 2016). Previous work tracking the fusion and endocytosis of Syb2 was performed by overexpressing Syb2‐pHluorin, which leads to abnormal subcellular distribution, including increased plasma membrane levels and suboptimal retrieval, primarily due to an imbalance in the ratio of Syb2 to its trafficking partner Synaptophysin‐1 (Gordon et al. 2016; Pennuto et al. 2003). Here, we demonstrate that EVs can be used to effectively and rapidly deliver Syb2‐pHluorin directly to axons and this exogenous Syb2‐pHluorin is incorporated into functional secretory vesicles, but only in a small subset and in very low levels. Thus, we next hypothesize that Syb2‐pHluorin delivered via EVs can be used to track Syb2 trafficking at presynaptic terminals in the absence of overexpression artifacts. To validate that there is no overexpression of Syb2 levels, we first estimated the number of Syb2‐pHluorin molecules (quanta or q) involved in each fusion event in response to single APs by fitting the distribution of amplitudes with a Gaussian mixture function (Figure 5A–C). This analysis showed that most detected vesicles carried between 1 and 4 copies of the probe (Figure 5C), which also suggests that only the smallest clusters observed via STORM are fusion‐competent, while the larger clusters may represent recycling or trafficking intermediaries. Considering that each synaptic vesicle carries ~70 copies of Syb2, the exogenous addition of 1–4 copies of Syb2‐pHluorin is well below endogenous levels and thus not overexpressed. We next investigated the fate of Syb2‐pHluorin after fusion using our previously validated methods (Chanaday and Kavalali 2018, 2021). We defined the kinetics of endocytosis as the time Syb2‐pHluorin resides at the cell surface, exposed to extracellular pH (7.4) before being retrieved, which is calculated by measuring the duration of the dwell time in fluorescence after fusion (Figure 5A). After endocytosis, the endosome or vesicle is acidified (pH ~ 5), quenching the fluorophore and causing a decay in fluorescence. By analyzing fusion events from individual vesicles, we can thus deconvolve endocytosis—dwell time—from acidification—decay time. Calculated dwell times pointed to very fast retrieval of Syb2‐pHluorin after fusion (Figure 5D,E). Fitting of logarithmic histograms with Gaussian mixture models predicted the presence of two populations with characteristic endocytic times of 700 ms and 1.5 s, corresponding to our previously reported ultrafast and fast endocytic pathways (Chanaday and Kavalali 2018, 2021). However, we did not detect slow endocytosis (> 5 s), suggesting this previously described mode of synaptic vesicle retrieval is either a very low‐probability event or a consequence of overexpression. Newly exocytosed Syb2 was previously proposed to undergo confined diffusion out of the presynaptic active zone (Gimber et al. 2015), with fusion being compensated by retrieval of preassembled clusters of synaptic vesicle and endocytic proteins at the periphery of the active zone (Del Signore et al. 2023; Hua et al. 2011; Imoto et al. 2022). Since Syb2‐pHluorin delivered via EVs resided mainly in internal organelles (Figure 4B), our system presented the ideal opportunity to track the destination of newly fused Syb2, that is, whether it stays at the surface or is rapidly retrieved, in the absence of confounding effects from surface signal. We defined the efficiency of retrieval as the ratio or fraction of the amplitude of the decay phase (peak back to baseline)–which depends on the number of Syb2‐pHluorin molecules that were retrieved–, respect to the amplitude of the fusion event (baseline to peak)–which corresponds to the number of fused Syb2‐pHluorin (Figure 5F). The majority (> 75%) of the fusion events showed quantal retrieval, that is, a ratio of 1.0 ± 0.2 (Figure 5F), suggesting that for most fusion events, all the fused probes were subsequently endocytosed. The remaining fusion events showed partial (14%) or no retrieval (8%; Figure 5F), indicating that, in some cases, the fused molecules are not instantly retrieved and remain at the plasma membrane, and a different portion of the membrane may undergo compensatory endocytosis. Taken together, by using EVs to sparsely deliver Syb2‐pHluorin to synaptic vesicles, we show that the main mode of Syb2 retrieval during low frequency activity consists of ultrafast and highly efficient endocytosis of newly fused Syb2.

## 4 Discussion

In the present study, we leverage our previous discovery that neurons secrete Syb2 via EVs to address two central questions in Syb2 trafficking: how and where Syb2‐containing EVs are internalized by neurons, and what happens to newly fused Syb2 molecules during low‐frequency synaptic activity. We demonstrate that Syb2‐containing EVs are incorporated throughout the neuron, including the somatodendritic and axonal compartments, via a Dynamin‐dependent pathway. This process is fast, peaking at 10–30 min and leading to the accumulation of exogenous Syb2‐pHluorin in internal acidic organelles. We next show that EVs can be used as a tool to sparsely deliver Syb2‐pHluorin to synaptic vesicles and track their activity‐driven fusion and endocytosis without overexpression artifacts. Using this approach, our results suggest that the main mode of Syb2 retrieval is ultrafast and highly efficient, supporting a model in which synaptic vesicle molecules remain clustered and are rapidly cleared from the plasma membrane during low frequency neuron activity.

### 4.1 Uptake of Syb2‐Containing EVs Into Neurons

Mounting evidence suggests that EVs are incorporated into various target cells primarily via endocytosis, including Clathrin‐mediated, Caveolin‐dependent, macropinocytosis, and phagocytosis (Escrevente et al. 2011; Feng et al. 2010; Fitzner et al. 2011; Mulcahy et al. 2014; Solana‐Balaguer et al. 2023; Tian et al. 2010). Here, we show that the Dynamin inhibitor Dyngo‐4a impeded the uptake of a specific subpopulation of neuronal EVs that carry the presynaptic protein Syb2, while the macropinocytosis inhibitor EIPA did not affect Syb2 EV endocytosis. Moreover, in the absence of Dynamin EVs fail to increase inhibitory neurotransmission, further supporting that this is the main route of incorporation of EV‐delivered Syb2 and it is essential for the downstream incorporation of Syb2 into functional synaptic vesicles. It is possible that other types of neuronal EVs that lack Syb2 follow alternative uptake and transport routes, as suggested previously in other cell types (Escrevente et al. 2011; Ginini et al. 2022; Mulcahy et al. 2014; Pedrioli and Paganetti 2020). In this context, our results investigating a specific subpopulation of neuronal EVs that carry Syb2 support the premise that different subtypes of EVs, depending on their molecular composition, are internalized via separate routes. We additionally show that EV uptake is very rapid, peaking at 10–30 min, and occurs throughout neuron compartments. The same Dynamin‐dependent endocytic mechanism is used by the somatodendritic and axonal compartments. Considering that we previously showed that secretion and uptake of Syb2‐containing EVs by neurons is independent of activity (Vilcaes et al. 2021), these results suggest there is a neuron‐wide constitutive EV‐uptake pathway that is surveilling the EVs in the environment at rest. Previous work in other neuron types highlighted that EV uptake seems to mainly occur in the neuron soma (Forero et al. 2024), which may facilitate the delivery of genetic materials and transcription factors to the nucleus. Our data show that Syb2‐containing EVs are also directly incorporated by axons and integrated into functional synaptic vesicles, thus influencing synaptic transmission; this further supports the premise that the subcellular location of EV internalization is crucial for fast delivery of its cargoes and for determining their functional effect. After internalization, Syb2 delivered via EVs follows an intra‐neuronal transport route that converges with that of Tf and LacCer and is segregated from macropinocytosis cargoes, showing that transport of EV components is not random but follows specific pathways that possibly mimic the normal transport mechanisms of endogenous molecules.

Our experiments show that Syb2‐containing EVs are internalized via a pathway dependent on Dynamin. Several Dynamin‐dependent endocytic pathways have been proposed in non‐neuronal cell types for EV uptake, including Clathrin‐mediated, Caveolin‐dependent, and Flotillin‐dependent endocytosis (Costa Verdera et al. 2017; Mulcahy et al. 2014). A previous report suggested that EV incorporation into cortical neurons occurs via Clathrin‐dependent endocytosis (Solana‐Balaguer et al. 2023). Additionally, neurons and neuronal EVs are rich in glycosphingolipids, which are retrieved from the surface mainly via Caveolin‐mediated endocytosis (Nusshold et al. 2013; Singh et al. 2003), so it is possible that Syb2‐containing EVs also use this pathway for their uptake. However, our results suggest low initial colocalization between Syb2‐containing EVs and both the Clathrin cargo Tf and the Caveolin cargo LacCer (Figure S3), pointing to segregated uptake pathways. An alternate route of interest is Flotillin‐dependent endocytosis. Flotillins have been suggested to regulate neuronal endocytosis of proteins like amyloid precursor protein and the dopamine transporter (Schneider et al. 2008; Cremona et al. 2011). Further, Flotillins are enriched in neuronal EV membranes (Vilcaes et al. 2021), though whether flotillin itself drives uptake of these EVs into neurons is unknown. Another interesting candidate for Dynamin‐dependent EV uptake is fast endophilin‐mediated endocytosis (FEME). Though the role of FEME in EV uptake in neuronal cells is poorly defined, a recent study established FEME as essential for endocytosis of plant‐derived dietary EVs in epithelial cells (Nguyen et al. 2025). In neurons, endophilin—along with its partner synaptojanin—is essential to neck formation during ultrafast endocytosis following synaptic vesicle fusion to the plasma membrane (Watanabe et al. 2018). Discerning among these uptake routes will be experimentally challenging, however, as pharmacology to block these pathways either lacks specificity or does not yet exist (Dutta et al. 2012; Guo et al. 2015; Szewczyk‐Roszczenko et al. 2023; Vercauteren et al. 2010).

### 4.2 Using EVs for Sparse Labeling of Synaptic Vesicles With Syb2‐pHluorin

Previous studies proposed several endocytic mechanisms to retrieve synaptic vesicles during neuron activity. One possibility is diffusion of synaptic vesicle molecules after full collapse fusion, and compensatory ultrafast or bulk endocytosis of a different portion of membrane in the periactive zone (Chanaday et al. 2019; Del Signore et al. 2023; Gimber et al. 2015; Li and Murthy 2001; Ogunmowo et al. 2023; Wu et al. 2014). Another option is that synaptic vesicle molecules remain clustered after fusion and are rapidly recaptured and retrieved (Alabi and Tsien 2013), a mechanism recently called “kiss‐shrink‐run” (Tao et al. 2025). Or a combination of both scenarios exists. Previous discrepancies arise from using different methodologies with variable spatial and temporal resolutions. Another caveat is the use of overexpression of pHluorin‐tagged proteins, which causes mislocalization, aberrant lateral diffusion, and defects in retrieval from the plasma membrane (Gordon et al. 2016; Pennuto et al. 2003). Here, we overcame these caveats by using EVs to deliver Syb2‐pHluorin to synapses. Our results show that fast and ultrafast endocytosis mediate most of the retrieval of synaptic vesicles during low‐frequency firing. The majority of these events retrieve all the fused molecules, indicating that synaptic vesicle components may remain clustered for fast recapture and retrieval. The remaining events show partial to no retrieval, suggesting the coexistence with diffusion and compensatory endocytosis, and agreeing with recent measurements using orthogonal methods (Tao et al. 2025). These results highlight the richness and complexity of mechanisms regulating synaptic vesicle recycling at synapses. This recycling diversity may have evolved to provide resilience such that synapses can continue to function even if specific endocytic mechanisms are compromised. This work opens the door for future applications of EVs for selective delivery of molecules to study transport and signaling in neurons, without overexpression artifacts.

A number of molecular mechanisms have been found to support synaptic vesicle recycling, including Clathrin‐mediated endocytosis and regeneration from endosomes, Dynamin‐dependent fast and ultrafast retrieval mediated by Formins and/or Synaptojanin and Endophilin, and Dynamin‐independent endocytosis (Afuwape et al. 2025; Chanaday et al. 2019; Gan and Watanabe 2018; Ivanova and Cousin 2022; Shin et al. 2021; Soykan et al. 2017). In the future, it would be interesting to elucidate whether the Dynamin‐dependent integration of Syb2‐containing EVs we demonstrate here is hijacking the same Dynamin‐dependent mechanism that endogenous synaptic vesicles utilize for their fast recycling at presynaptic terminals during neuron activity (Cheung and Cousin 2019; Imoto et al. 2024; Shi et al. 2022).

### 4.3 The Roles and Applications of EVs in the Nervous System

EVs participate in all stages of neurodevelopment, from aiding in the proliferation and differentiation of neural precursor cells to modulating synapse formation and function (Bahram Sangani et al. 2021; Zhang, Lin, et al. 2021). A recent study determined that EVs traffic molecular cues relevant to brain development (Forero et al. 2024). Considering the crucial role of Syb2 in neuron and synapse development (Bogue et al. 2023; Guzikowski et al. 2025; Salpietro et al. 2019), our findings suggest Syb2 may be one of these EV cargoes. In adult neurons, neuron‐derived EVs exert trophic effects that promote survival, augment synaptic transmission and facilitate dendritic structural remodeling thus controlling plasticity and brain function (Lee et al. 2018; Solana‐Balaguer et al. 2023; Vilcaes et al. 2021; Zhang, Zhang, et al. 2021). Our findings suggest that these neuron‐derived EVs can be incorporated throughout all neuronal compartments in a constitutive manner, thus ensuring the effective delivery of EV components independently of the metabolic state of the target neuron. This could be particularly relevant in the context of aging, where Syb2 levels are reduced and this is sufficient to cause cognitive impairment (Miller et al. 2026; Orock et al. 2020). Thus, the exchange of Syb2 via EVs could improve neuron function during aging, including in those neurons with reduced activity due to loss of endogenous Syb2. In recent years, considerable advances have been made in engineering EVs to optimize cargo loading and brain targeting while reducing liver, lung and spleen toxicity, improving blood–brain barrier permeability, reducing their immunogenicity and improving their endocytosis and beneficial effects to treat neurological disorders (Fekri et al. 2019; Hu et al. 2025; Lerussi et al. 2025; Nieland et al. 2023; Saint‐Pol et al. 2020). In this context, engineering EVs for targeted delivery of Syb2 and other trophic signals across the blood–brain barrier or developing targeted treatments to enhance Syb2‐containing EV endocytosis are both feasible and promising therapeutic avenues. A current challenge to achieve this goal is the limited scalability of EV production and the high batch‐to‐batch variability in EV molecular composition (Herrmann et al. 2021; Li et al. 2019), which may impact effectivity and increase the risk of unwanted side effects.

## Author Contributions

**Victoria Hannett:** methodology, investigation, validation, data curation, visualization, writing – original draft, writing – review and editing, formal analysis. **A. Alejandro Vilcaes:** conceptualization, methodology, data curation, investigation, validation, formal analysis, resources, visualization, writing – original draft, writing – review and editing. **Siewert Hugelier:** conceptualization, software, formal analysis, writing – review and editing, investigation. **Qing Tang:** conceptualization, software, formal analysis, investigation, writing – review and editing. **Melike Lakadamyali:** writing – review and editing, resources, funding acquisition, conceptualization, supervision. **Natali L. Chanaday:** conceptualization, investigation, funding acquisition, writing – original draft, writing – review and editing, methodology, validation, visualization, software, formal analysis, project administration, data curation, supervision, resources.

## Funding

This work was supported by the Margaret Q. Landenberger Research Foundation. National Institute of General Medical Sciences, GM152111, GM136511.

## Conflicts of Interest

The lead author, Natali L. Chanaday, is a handling editor at the Journal of Neurochemistry.

## Supporting information


**Figure S1:** Characterization of EVs secreted from dissociated rat neurons.
**Figure S2:** EV‐delivered Syb2 transport converges with Tf and LacCer in the somatodendritic compartment.
**Figure S3:** Neuronal EVs have no effect on spontaneous inhibitory neurotransmission in the absence of all Dynamin isoforms.

## Data Availability

All data and resources generated during this work are available upon reasonable request to the corresponding author (NLC—natali.chanaday@pennmedicine.upenn.edu).
