# Supplementary material for: Synaptobrevin‐2 Containing Extracellular Vesicles Are Rapidly Incorporated Into Mammalian Neurons via a Dynamin‐Dependent Pathway
Source: J Neurochem. 2026 Sep 11;170(9):e70551. doi: 10.1111/jnc.70551 (PMC13563309; doi:10.1111/jnc.70551)
Supplement: Supplementary file 1 [file JNC-170-e70551-s001.pdf]

Supplementary Material for:

Sparse delivery of Synaptobrevin-2 to mammalian neurons using extracellular vesicles

Victoria Hannett[1,2], A. Alejandro Vilcaes[1,3], Siewert Hugelier[1], Qing Tang[4], Melike Lakadamyali[1,5], Natali L. Chanaday[1,6]

[1] Department of Physiology, Perelman School of Medicine, University of Pennsylvania, Philadelphia, PA 19104, USA

[2] Master of Biotechnology Program, School of Engineering and Applied Science, University of Pennsylvania, Philadelphia, PA 19104, USA

[3] Consejo Nacional de Investigaciones Científicas y Técnicas (CONICET), Universidad Nacional de Córdoba, Córdoba X5000HUA, Argentina

[4] Department of Biochemistry, Institute of Agriculture and Natural Resources, College of Arts and Sciences, University of Nebraska-Lincoln, Lincoln, NE 68588, USA

[5] Penn Muscle Institute and Epigenetics Institute, University of Pennsylvania, Philadelphia, PA 19104, USA

[6] Department of Neuroscience, Perelman School of Medicine, University of Pennsylvania, Philadelphia, PA 19104, USA

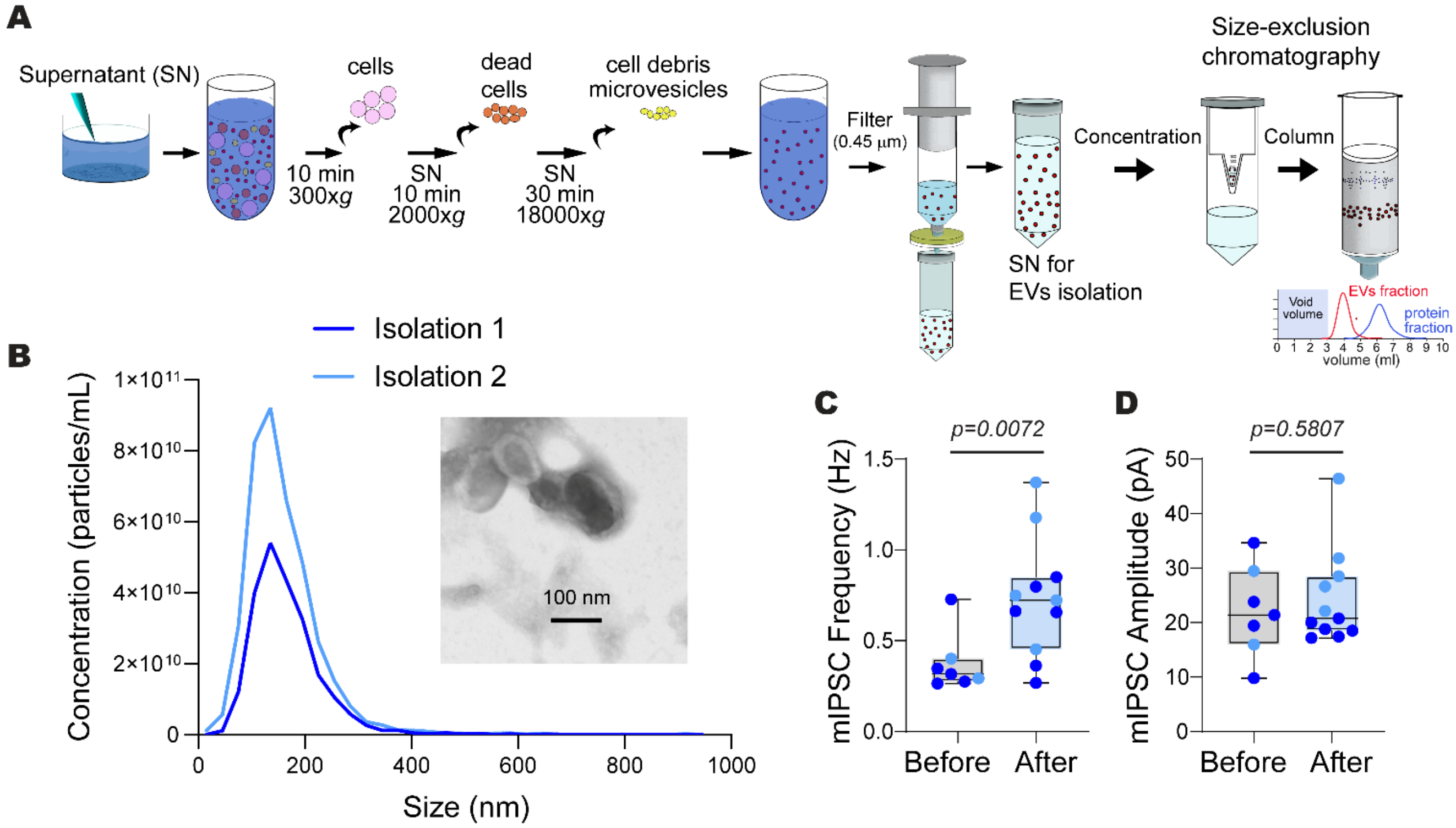

Figure S1. Characterization of EVs secreted from dissociated rat neurons.

A. Schematic representation of EVs isolation procedure. Supernatant from dissociated hippocampal neurons is first cleared from debris by serial centrifugations and then EVs are isolated by size-exclusion chromatography (see details in Methods section). **B.** Neuronal EVs consist of a homogeneous population of small EVs with diameter ~120-140 nm as evidenced by Nanoparticle Tracking Analysis (NTA) and negative stain electron microscopy (inset). **C-D.** Frequency and Amplitude of miniature inhibitory postsynaptic currents (mIPSC) before and 25-45 minutes after addition of neuronal EVs. Data from two independent EV isolation experiments. For C and D, each dot represents one recorded neuron, groups compared using unpaired t-test.

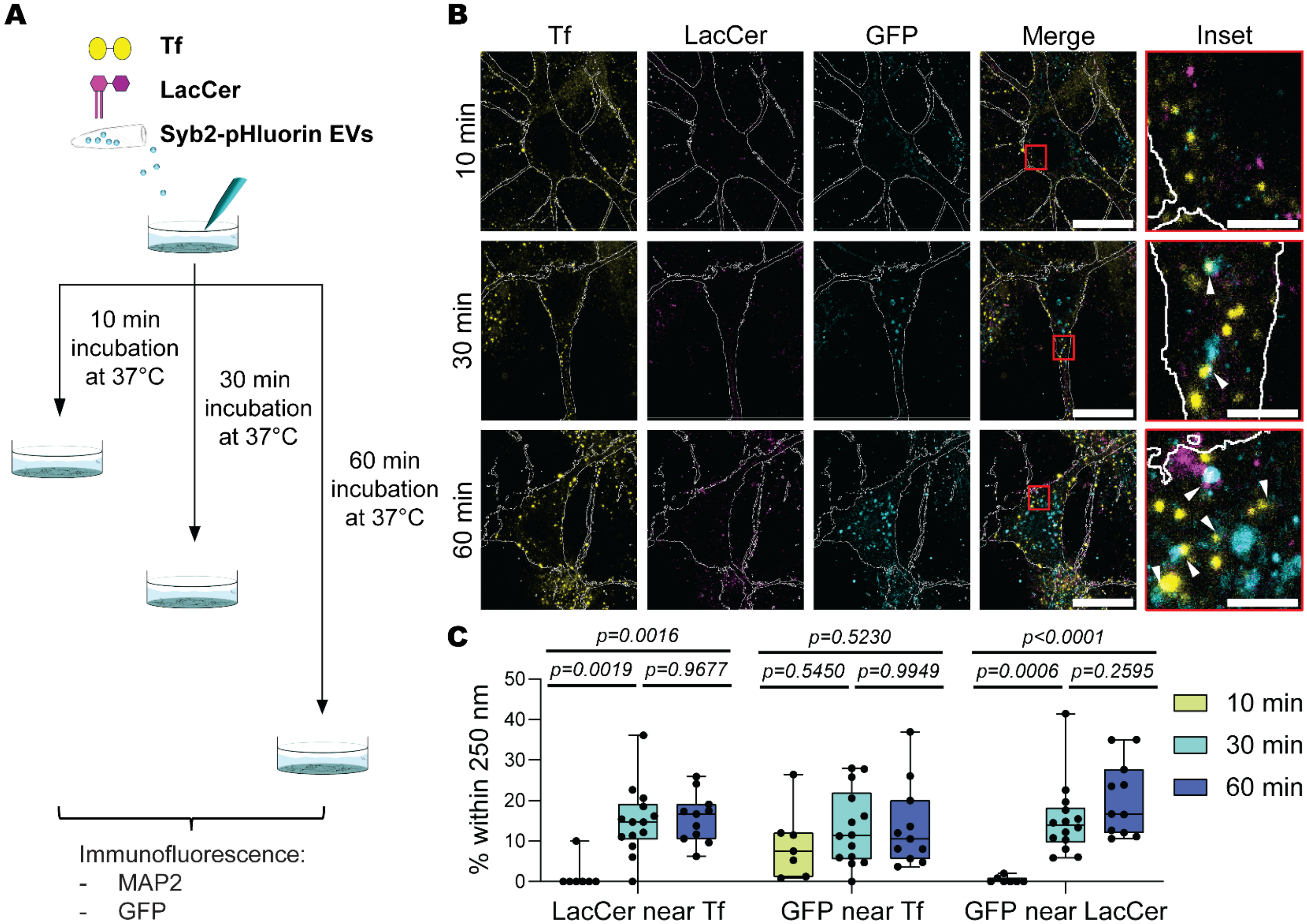

Figure S2. EV-delivered Syb2 transport converges with Tf and LacCer in the somatodendritic compartment.

A. Experimental design. **B.** Representative confocal images of neuronal cell bodies immunofluorescently labeled with TF (yellow), LacCer (magenta) and anti-GFP (cyan). MAP2 staining was used to create masks around the somatodendritic compartment for analysis (shown in the white outlines). Z-stacks of images are shown, presented as the sum-of-slices projection of the volumetric data. White scale bars = 10 µm. Inset white scale bars = 1 µm **C.** Percentage of fluorescent objects within 250 nm across two fluorescent labels, following all incubation times. Each point represents one neuronal cell body, data from 2 independent cultures, analyzed by two-way ANOVA, individual p-values from Tukey's multiple comparisons test are displayed in the figure.

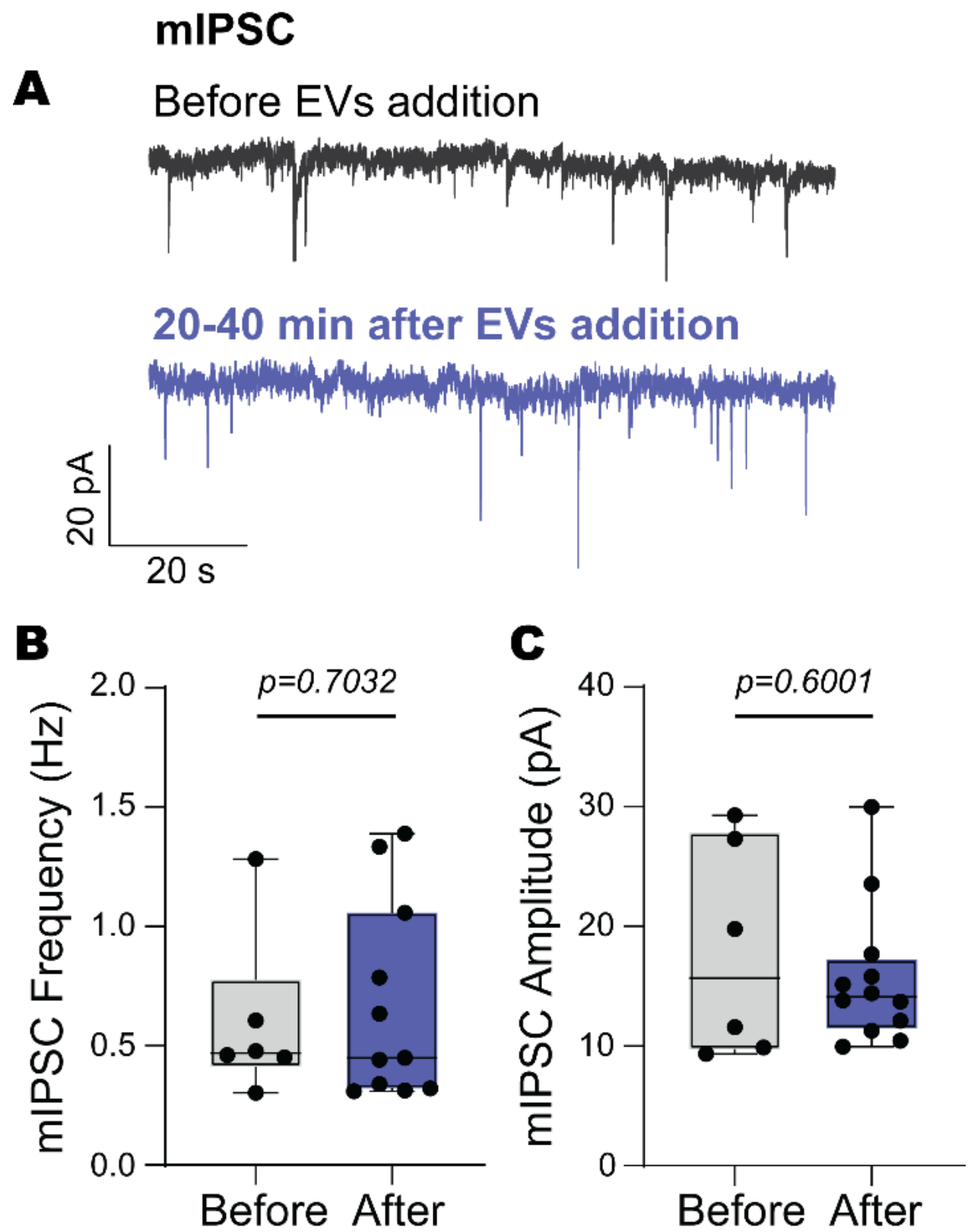

Figure S3. Neuronal EVs have no effect on spontaneous inhibitory neurotransmission in the absence of all Dynamin isoforms.

A. Representative miniature inhibitory postsynaptic current (mIPSC) recordings. **B-C.** mIPSC Frequency and Amplitude before and 25-45 minutes after addition of neuronal EVs. Data from two independent Dynamin triple knock-out cultures, each dot represents one recorded neuron, groups compared using unpaired t-test.
